# The New Operating Department of the London Hospital

**Published:** 1902-05-31

**Authors:** 


### The Institutional Workshop.

### THE NEW OPERATING DEPARTMENT OF THE LONDON HOSPITAL.

### OPENING BY THE LORD MAYOR.

A large concourse of people assembled at the London Hospital on Thursday afternoon (22nd inst.), when the new operating department was opened with civic honours. The Lord Mayor, who was accompanied by the Lady Mayoress. Mrs. Dent, the Sheriffs, the Sword-bearer, the Mace-bearer, and the City Marshal, was received by Mr. John Henry Buxton (Treasurer), the Hon. Sydney Holland (Chairman), Dr. Stephen Mackenzie (Senior Physician), Mr. C. Mansell-Moullin (Surgeon), and Mr. G. Q. Roberts (House Governor), and was conducted to the library of the Medical College. The room was already crowded with visitors, and the students in the gallery cheered the Lord Mayor with great enthusiasm.

Mr. J. H. Buxton, in opening the proceedings, said there had been a time when the attendances of the Lord Mayor on the House Committee were higher than those of himself as chairman. This was a City Hospital; and it was only 150 years ago that it was moved from Aldgate to the country lanes and green fields of Whitechapel. It still stood on a freehold of City land, and paid its rent with great regularity. Mr. Buxton concluded by reading a letter from Mr Jonathan Hutchinson, whose name called forth applause from the gallery, regretting that he was unable to meet the Lord Mayor at the hospital.

#### The Lord Mayor's Speech.

The Lord Mayor, who was warmly applauded, said it was not a quarter of a century since he first took his place on the house committee under Mr. J. H. Buxton's chairmanship, when Mr. Thomas F. Buxton (his father) was the treasurer. The name of Buxton would always be held in veneration, regard, and respect. It was with feelings akin to amazement that he saw what progress had been made at the hospital within a very short time. The Lord Mayor then gave figures showing the enormous increase since 1871 in the number of patients treated (6,303 in 1871, 13,364 in 1901). Just at the moment when help was most urgently wanted, he said, the Grocers' Company—to which he had the honour to belong—stepped in and provided a fund for building the Grocers' wing. While the necessary accommodation for the ever-growing number of patients was being found, other developments had had to stand on one side; but among other agencies somewhat outside the actual wards was the absolutely necessary one of an adequate theatre. Every important hospital should be thoroughly equipped, and should give its staff the opportunity of using their skill to the full. During the past 30 years, the term of residence, owing to improved surgical methods, had been reduced from 33 to 18 days. This was not only a great economic gain to the community in general, but it increased the hospital's power of doing good. The number of surgical cases had grown from 3,417 in 1871 to 7,503 in 1901; thus the need for more theatre accommodation had grown up, and an anonymous donor had arisen to give it. A suite of rooms had been built as complete and efficient as skill and forethought could contrive, at a cost of £13,000. In opening these rooms, he could not help expressing the gratitude everyone felt towards the donor, who desired his name to be kept secret; for his own part he had no conception of who he might be, but his gratitude was none the less hearty and sincere because it could not be conveyed to him. One of the greatest honours this country possessed was that the hospitals were kept up by voluntary contributions, the outcome of the love and sympathy of the people; but there was always one source of great anxiety to those who administered their affairs, viz., the difficulty of paying their way. The only way to do this was to let the benevolent and generous public know that it required a very large amount of money to carry on the work efficiently and thoroughly. The expenses of this hospital were necessarily very large—last year £83,000 was spent, including maintenance and administration, and he asked his old friends the press, who never shrank from helping any charitable work, to let the British public know the needs of this institution, which was doing a work, so far as he knew, unparalleled by any other in the world. He prayed that a blessing might rest on the work and on all who had a share in it.

#### The Anonymous Donor.

The Hon. Sydney Holland, after thanking the Lord Mayor most warmly for his speech, said he envied his power of doing good, and the facile pen with which he wrote begging letters to the *Times*. Perhaps when his term of office was over he would start a school for hospital chairmen. He wanted to put the increase in the work of the hospital in an even more striking way than the Lord Mayor had done, and to say that unless the bandages were taken off within ten days someone would want to know the reason why. Operations were not to be called horrors now, for one was hardly in the fashionable world if one had not undergone surgical treatment. The hospital was proud of its surgical staff, and it was as much as the house committee's life was worth to refuse them anything. Great credit was due to the nurses, and to that woman who for 20 years had devoted herself to bringing the nursing to perfection. Speaking of the new surgical theatres, Mr. Holland said that some three years ago, a gentleman who had been introduced by Mr. Cohen was going round the wards of the London Hospital. In the course of his visit he noticed some of the patients sitting in red dressing-gowns, and on inquiry was told they were waiting their turn for operation, but that owing to the fact that there was only one operating theatre available, it was quite likely that they would be disappointed, and would have to wait until the following week. Struck by the unnecessary suffering this involved, he asked what it would cost to provide a sufficient number of operating theatres, and was told £13,000. This sum he generously and at once gave, attaching only two conditions to his gift; firstly, that it should be open to all denominations; and secondly, that his name should be kept secret. The striking necessity for this addition was shown by the great increase in the number of major operations performed at the hospital:—In 1881 there were 420; in 1891, 1,144; in 1901, 2,711.

Mr. Mansell-Moullin described the new department as one that had not its equal in the United Kingdom; it had been designed after a careful comparison of the best theatres at home and abroad. The surgical staff were very grateful to the house committee for all these things; the theatre alone would have been much, but there was a great deal besides.

After prayer by the Bishop of Stepney, the Lord Mayor went to visit the new department, and the visitors were entertained with refreshments, and music by the Lawler Quintette in Gloucester Ward.

#### Description.

The new operating department, which was visited by T.R.H. the Prince and Princess of Wales on March 20th, is on the third floor of the old front block, on the site formed by demolishing the attics of the old operating and clinical theatres. The department consists of a theatre, four operating rooms and three anæsthetising rooms, all of which face north, and so get a good diffused light just as a studio does. There are also four recovery rooms, or small wards, five operation wards, two sisters' rooms, a nurses' room, waiting room, theatre-superintendent's room, two attendants' rooms, instrument and sterilising rooms, surgeons' rooms with two examining rooms and lavatory, and dressers' lavatory. When the east end is completed there will be some more small wards on the fourth floor. The wide central corridor is approached by two large staircases and by two new lifts.

The operating rooms are about 22 by 24 feet. The floors are paved with terazzo, and the walls and ceilings are lined with opaline tiles in light blue or green, and white. The artificial light is supplied by powerful electric lamps, one cluster of which is equal to 80 candle-power. Single pendants are placed round the room, and these can be carried to the table if necessary. Near the head of the table is a pendant of plugs; these are for working surgical motors, cauteries, cystoscopes, hand-lamps, etc. Three emergency lamps are fixed under this, for use in case of a breakdown in the main supply; the generator which charges the accumulators is driven by a current from the mains of the Borough Council. Ventilation is supplied by a modification of the Plenum system, and the windows are air-tight. The air comes from the garden, through a glazed tunnel. After passing through a canvas screen and cotton-wool filters, it is warmed by steam coils; and the warm and cold air come in separate tubes by high pressure, and are diffused by a grating. The modification in the system consists of an electric fan which sucks out the used air. The amount of air admitted is regulated by a small handle in the wall; and the air can be cooled or heated to the required temperature in three minutes. A steam jet is used to damp the air and free it from dust.

All the fixed shelves, etc., are of marmorite.

The lavatory arrangements have been made to special sketches, by Messrs. Marshall and Woods. A row of sinks is supplied with brass taps by which either a spray or a stream of hot, cold, or tepid water is obtained; there is no plug to work with the feet, the taps being so arranged that they can be turned off with the elbows. These elbow-taps were copied from some in use at Dundee. The pipes are straight, so as to be easily cleaned out with a brush. All the water is boiled, and each day's cold water supply is cooled in a special apparatus.

With regard to furniture, a "routine list" was prepared by the committee of the hospital, and each surgeon has been free to order from any maker he might prefer. A fascinating aluminium stool for the anæsthetist forms part of one set; this runs on castors with ball-bearings, with great ease, and there are no corners to collect dust.

The particular form of operation table has not yet been finally settled, but whatever kind is chosen, it will be simple in make.

Only one of the operating rooms, the one described as the theatre, has a students' gallery. The difficulty at the London Hospital is, not a lack of sufficient operations for the students, but sufficient students as dressers, and therefore one galleried-room has been thought enough. The entrance to this is independent of the entrance to the theatre itself. The gallery is of marble, with teak seats and gun-metal rails.

As an instance of the care given to details, we observed, during our preliminary visit of inspection, a small lavatory in the wall of the students' gallery; this is for use after specimens have been passed round for examination.

